# P﻿raktische Anleitung zur Implantation nicht-transvenöser ICD-Systeme

**DOI:** 10.1007/s00399-024-01042-w

**Published:** 2024-08-21

**Authors:** David Duncker, Karolin Albert, Andreas Rillig, Philipp Sommer, Christian-Hendrik Heeger, Melanie Gunawardene, Sascha Rolf, Henning Jansen, Heidi Estner, Till Althoff, Tilman Maurer, Roland Tilz, Leon Iden, Victoria Johnson, Daniel Steven

**Affiliations:** 1 Hannover Herzrhythmus Centrum, Klinik für Kardiologie und Angiologie, Medizinische Hochschule Hannover, Carl-Neuberg-Str. 1, 30625 Hannover, Deutschland; 2 Universitäres Herzzentrum Hamburg, Universitätsklinikum Eppendorf Hamburg, Hamburg, Deutschland; 3 Klinik für Elektrophysiologie und Rhythmologie, Herz- und Diabeteszentrum NRW, Ruhr-Universität Bochum, Medizinische Fakultät der Universität Bielefeld, Bad Oeynhausen, Deutschland; 4 Department für Rhythmologie, Asklepios Klinik Hamburg Altona, Hamburg, Deutschland; 5 Klinik für Kardiologie und internistische Intensivmedizin, Asklepios Klinik St. Georg, Hamburg, Deutschland; 6 Klinik für Innere Medizin mit Schwerpunkt Kardiologie, DRK Kliniken Berlin Westend, Berlin, Deutschland; 7 Elektrophysiologie Bremen, Bremen, Deutschland; 8 Medizinische Klinik und Poliklinik I, LMU Klinikum München, München, Deutschland; 9 Arrhythmia Section, Department of Cardiology, CLINIC Barcelona University Hospital, Barcelona, Spanien; 10 CardioMed Hamburg, Hamburg, Deutschland; 11 Klinik für Kardiologie und internistische Intensivmedizin, Asklepios Klinik Nord, Hamburg, Deutschland; 12 Klinik für Rhythmologie, Universitäres Herzzentrum Schleswig-Holstein Lübeck, Lübeck, Deutschland; 13 Deutsches Zentrum für Herzkreislaufforschung (DZHK), Partner Site Hamburg/Kiel/Lübeck, Lübeck, Deutschland; 14 Herz- und Gefäßzentrum Segeberger Kliniken, Bad Segeberg, Deutschland; 15 ZIM – Med. Klinik 3 – Kardiologie, Angiologie, UHF – Universitäres Herz- und Gefässzentrum, Frankfurt, Deutschland; 16 Abteilung für Elektrophysiologie, Herzzentrum der Uniklinik Köln, Köln, Deutschland

**Keywords:** Implantierbarer Kardioverter-Defibrillator, Plötzlicher Herztod, Extravaskuläre Systeme, Subkutaner ICD, Ventrikuläre Tachykardien, Extravaskulärer ICD, Implantable cardioverter-defibrillator, Sudden cardiac death, Extravascular systems, Subcutaneous ICD, Ventricular tachycardias, Extravascular ICD

## Abstract

Als Alternative zu transvenösen ICD-Systemen sind aktuell zwei nichttransvenöse ICD-Systeme verfügbar: Der seit einigen Jahren etablierte subkutane ICD (S-ICD) verfügt über eine prästernale Elektrode, die subkutan implantiert wird und die eine Schockfunktion sowie in begrenztem Umfang auch eine Post-Schock-Stimulation bietet. Außerdem ist in Europa seit 2023 der extravaskuläre ICD (EV-ICD) erhältlich, welcher ebenfalls ohne transvenöse Elektroden auskommt und die Möglichkeit bietet, Patienten mit einer antibradykarden und antitachykarden Stimulation in Kombination mit einer herkömmlichen ICD-Funktion zu versorgen. Bei diesem Device erfolgt die Elektrodenimplantation substernal. Erste Implantationsergebnisse sind im Hinblick auf Sicherheit und Effektivität vielversprechend. Beide Systeme umgehen einige mögliche Komplikationen transvenöser Elektroden. Dieser Artikel soll eine praktische Übersicht der Implantationsschritte und möglicher Komplikationen geben.

## Abstract (en)

As an alternative to transvenous ICD systems, two non-transvenous ICD systems have been established in recent years: The subcutaneous ICD (S-ICD), which has been established for several years, has a presternal electrode that is implanted subcutaneously and offers a shock function and, to a limited extent, post-shock pacing. In addition, the extravascular ICD (EV-ICD) has been available in Europe since 2023 which does not require transvenous electrodes and offers the option of providing patients with antibradycardic and antitachycardic stimulation in combination with a conventional ICD function. The lead of this device is implanted substernally. Initial implantation results are promising in terms of safety and effectiveness. Both systems avoid possible complications of transvenous electrodes. This article provides practical guidance for the implantation technique and possible complications.

## Hintergrund

Zur Prävention des plötzlichen Herztods aufgrund ventrikulärer Tachyarrhythmien stellen transvenöse implantierbare Kardioverter-Defibrillator-Systeme eine seit Jahren etablierte Therapie dar [2, 8]. Die transvenöse Elektrode ist eine bekannte Ursache für Komplikationen im Langzeitverlauf einer ICD-Therapie [16, 24]. Der subkutane ICD (S-ICD) ermöglicht eine ICD-Therapie ohne Zugang zum Gefäßsystem. Langzeitanalysen zeigen gute Ergebnisse hinsichtlich Sicherheit und Effektivität. Insbesondere bei jungen Patienten, die keinen Stimulationsbedarf haben, hat sich der S‑ICD zu einem etablierten Device entwickelt [3, 6, 18, 19]. Auch nach Infektionen transvenöser Systeme oder bei vaskulären Zugangsproblemen spielt der S‑ICD eine Rolle. Was bislang fehlte, ist die Möglichkeit zur antitachykarden Stimulation (ATP) oder Stimulation zur Prävention einer Asystolie ohne Implantation eines transvenösen oder zusätzlichen elektrodenlosen Systems [6, 13]. Mit dem extravaskulären ICD (EV-ICD) besteht die Option, Patienten mit einem System zu versorgen, das all diese Therapieformen in Kombination mit der Schockfunktion ermöglicht [28]. Eine Übersicht über die unterschiedlichen Systeme ist in Tab. 1 zu finden.

**Tab. 1:** Vergleich der unterschiedlichen ICD-Systeme. (Adaptiert nach [1, 28, 36])

Transvenöser ICD vs. S‑ICD vs. EV-ICD			
	*TV-ICD*	*S‑ICD*	*EV-ICD*
Aggregatposition	Präpektoral	Subkutan/intermuskulär (mittlere Axillarlinie links)	Subkutan (mittlere Axillarlinie links)
Elektrodenposition	Intrakardial	Subkutan	Substernal
Aggregatgewicht (g)	79	130	77
Batterielebensdauer in Jahren	10,8	5,7–8,7	11,7
ATP	Vorhanden	Nicht vorhanden	Vorhanden
Antibradykarde Stimulation	Vorhanden	Nicht vorhanden	Asystolie-Backup
Post-Schock-Stimulation	Vorhanden	Vorhanden	Vorhanden
Max. Defibrillationsenergie in Joule	40	80	40

## Der S-ICD

### Aufbau und Funktion

**Abb. 1:**
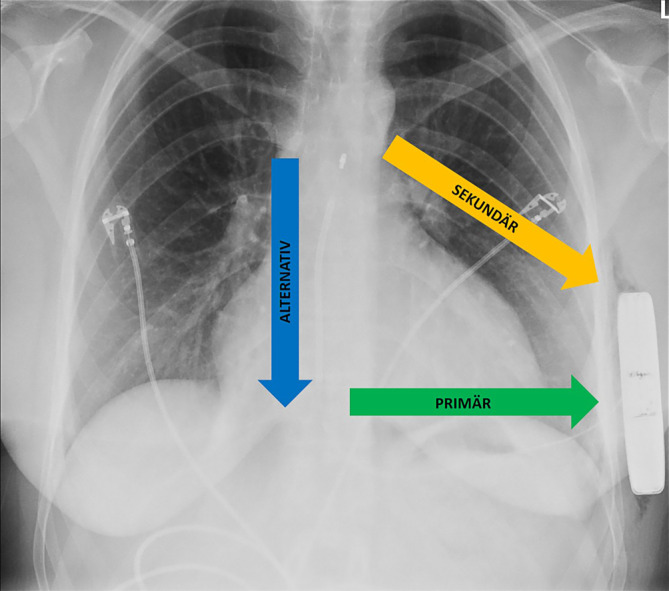
Der Aufbau eines S‑ICD anhand eines postoperativen Röntgen-Thorax von anterior-posterior. Schematisch sind die verschiedenen Wahrnehmungsvektoren eingezeichnet (*grün* primär, *gelb* sekundär, *blau* alternativ )

Beim S‑ICD handelt es sich um ein seit Jahren etabliertes Device, welches immer häufiger implantiert wird und eine breite Datenlage bezüglich Sicherheit der Implantation und Anwendung hat [3, 6, 21]. Da dieses Device subkutan implantiert wird, besteht auch hier die Möglichkeit, zahlreiche Risiken der transvenösen Systeme zu umgehen. Die S‑ICD-Elektrode besteht aus einer 45 cm langen Sonde, welche zwei Sensing-Elektroden mit einer dazwischenliegenden Schock-Coil von 8 cm aufweist (Abb. 1 und 2; [37]). Die Elektrode wird prästernal angelegt und verläuft dann zur linken vorderen Axillarlinie, wo das Aggregat in einer subkutanen oder intermuskulären Tasche liegt [14]. Die Wahrnehmung kann über drei unterschiedliche Vektoren erfolgen, zwischen dem Aggregatgehäuse und dem proximalen bzw. distalen Sensor liegen der primäre und der sekundäre Vektor (Abb. 1). Ferner gibt es zwischen beiden Ringen den sog. alternativen Vektor, über den ebenfalls ein Sensing erfolgen kann [17]. Eine Schockabgabe erfolgt entweder über den Standardvektor (Schock-Coil zu Aggregat) oder in umgekehrter Richtung. Im Anschluss an eine Schockabgabe ist für 30 s eine Post-Schock-Stimulation über den gleichen Vektor wie für die Schockabgabe (Frequenz von 50/min mit 200 mA bis zu 30 s) möglich [4]. Aufgrund der größeren Entfernung zum Myokard ist zur erfolgreichen Defibrillation eine höhere Energiemenge notwendig, sodass Schockabgaben mit bis zu 80 J erfolgen können [17]. Mithilfe der sog. SMART-Pass-Funktion besteht zudem die Möglichkeit, Artefakt-Oversensing zur reduzieren [17, 25].

**Abb. 2:**
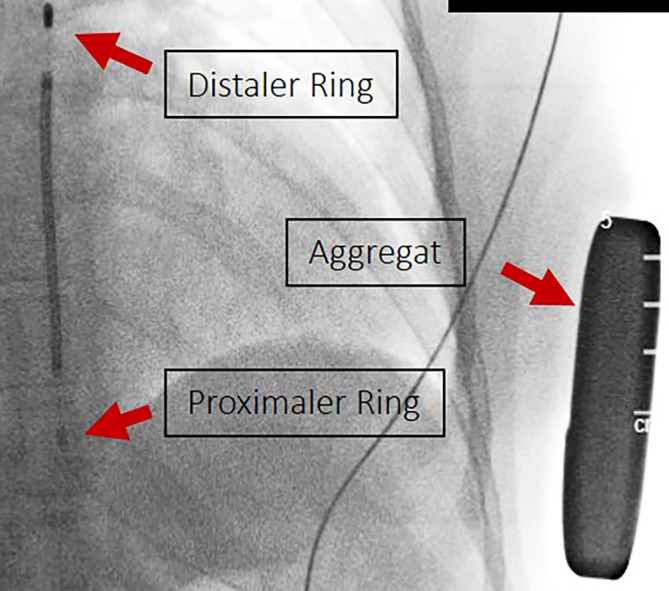
Präoperatives Anlegen des S‑ICD unter Röntgendurchleuchtung zur Planung der Implantation und Markierung der Orientierungspunkte. Deutlich erkennbar sind der distale und proximale Ring (Sensing-Elektroden), die Schock-Coil sowie das Aggregat in der linken Axillarlinie

### Patientenselektion und Operationsvorbereitung

Der S‑ICD kommt bei Patienten in Frage, die zwar eine Indikation zur ICD-Implantation aufweisen, jedoch keine Bradykardien mit dauerhaftem Stimulationsbedarf oder eine Indikation zur kardialen Resynchronisationstherapie haben. Eine Programmierung der VT-Zonen ist beim S‑ICD ab 170/min möglich, sodass Patienten nach stattgehabten ventrikulären Tachykardien (sekundärprophylaktische Indikation) häufig keine idealen Kandidaten darstellen [4, 17]. Patienten nach Sternotomie können grundsätzlich mit einem S‑ICD versorgt werden. Im Rahmen der Implantation sollte lediglich beachtet werden, dass die Sensing-Elektroden nicht in Kontakt mit den Sternalcerclagen kommen. Im Vorfeld erfolgt ein Screening mittels Oberflächen-EKG und dem Programmiergerät des Herstellers im Liegen und Sitzen, im Rahmen dessen alle drei Sensing-Vektoren auf suffiziente Wahrnehmung geprüft werden [31–35]. Ausreichend ist ein adäquates Sensing auf einem der drei Vektoren. Das Screening kann rechts- und linkssternal erfolgen und kann bei Unterschieden im Screeningerfolg bei der Implantation berücksichtigt werden.

### Implantation

Die Implantation eines S‑ICD erfolgt zumeist unter Lokalanästhesie und Analgosedierung. Möglich ist jedoch auch die Implantation im Rahmen einer Allgemeinanästhesie oder die Kombination aus Lokalanästhesie und lokaler Nervenblockade [4, 29]. Die perioperative Vorbereitung und Etablierung des Monitorings und der Defibrillationsbereitschaft sind obligatorisch [20]. Ebenso sollte eine präoperative Markierung der anatomischen Orientierungspunkte (Elektroden- und Aggregatposition unter Durchleuchtung, Proc. xiphoideus, mittlere Axillarlinie) erfolgen, da die spätere Elektroden- und Aggregatposition eine große Relevanz für das Sensing und Effektivität der Schockabgabe haben (Abb. 2).

Zunächst erfolgt eine Inzision paraxiphoidal rechts oder links auf Höhe des Xiphoids mit etwa 2 cm Länge. Dann wird auf die Faszie des M. rectus abdominis präpariert, auf der später der Sleeve der Elektrode fixiert werden wird. Anschließend wird die sternale Tunnelierung vorgenommen. Bei der Positionierung sollten auch etwaige Unterschiede im EKG-Screening (rechts- oder linkssternal) sowie der Praetorian Score berücksichtigt werden [23]. Die Tunnelierung sollte unmittelbar auf dem Periost des Sternums erfolgen, da eine Positionierung subkutan im Unterhautfettgewebe die DFT negativ beeinflussen kann. Die Messwerte für die Schockimpedanz sollten nach Herstellerangaben zwischen 25 und 110 Ω liegen. Im Anschluss wird die Aggregattasche intermuskulär zwischen M. serratus und M. latissimus dorsi präpariert [7]. Nun kann die Elektrode nach lateral in die Tasche tunneliert und mit dem Aggregat konnektiert werden. Das Aggregat sollte weit dorsal liegen und mit doppelten Annähten in der Tasche fixiert werden. Auf Luftfreiheit entlang der Elektrode und in der Aggregattasche sollte geachtet werden, da größere Lufteinschlüsse die erfolgreiche Defibrillation behindern können. Nach schichtweisem Wundverschluss erfolgt üblicherweise eine ICD-Testung. Bei nicht erfolgreicher Defibrillation kann eine Repositionierung der Elektrode und/oder des Aggregats oder Ausstreichen der Lufteinschlüsse hilfreich sein.

Vor Entlassung des Patienten sollte ein postoperatives Röntgen-Thorax in zwei Ebenen zu Dokumentation der Elektrodenlage sowie eine Device-Kontrolle des implantierten Systems erfolgen. Bei komplikationslosem Verlauf ist eine Entlassung am Folgetag möglich. Bis zur Device-Abfrage ist ein telemetrisches Monitoring der Patienten sinnvoll.

### Komplikationen

Bei Patienten, die mit einem S‑ICD versorgt wurden, zeigen sich geringe Komplikationsraten. Insbesondere sondenassoziierte Komplikationen sind signifikant seltener als bei transvenösen Systemen [6, 18]. Bei einer S‑ICD-Implantation sind die häufigsten Komplikationen Blutungen in die Aggregattasche sowie Infektionen, wobei diese im Vergleich mit transvenösen Systemen seltener zu systemischen Manifestationen und Sepsis führen [18]. Doch auch Sondendislokationen und Schwierigkeiten beim Sensing sind bekannte Komplikationen, insbesondere das T‑Wellen-Oversensing kann zu inadäquaten Therapien führen [18, 22]. Seitens des Herstellers wurde hierzu die sog. SMART-Pass-Funktion entwickelt, welche mithilfe eines Frequenzfilters von 9 Hz T-Wellen-Oversensing vermeiden soll [25, 27]. Bei Komplikationen im Kurz- oder Langzeitverlauf ist der Wechsel auf ein transvenöses System möglich. Ferner kann die Umstellung auf ein transvenöses System dann sinnvoll sein, wenn ein Stimulationsbedarf entsteht oder eine CRT-Indikation vorliegt.

## Der EV-ICD

### Aufbau und Funktion

**Abb. 3:**
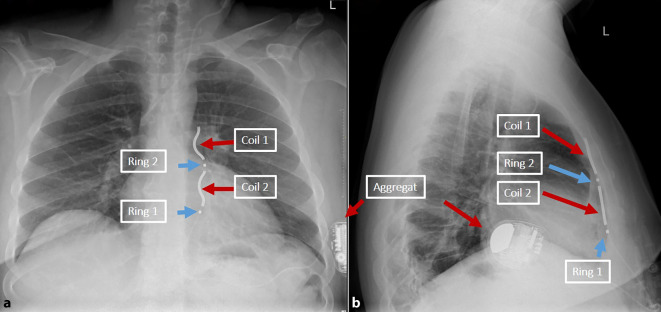
Aufbau eines EV-ICD anhand eines postoperativen Röntgen-Thorax in zwei Ebenen: **a** a.-p., **b** lateral. Die Elektrode weist je zwei Ringe zur Stimulation (Ring 1 und 2, *blaue Pfeile*) und zwei Schock-Coils (Coil 1 und 2, *rote Pfeile*) auf. In der vorderen linken Axillarlinie ist das Aggregat zu erkennen

Der EV-ICD besteht aus einem ICD-Aggregat in Form in Größe eines konventionellen Einkammer-ICD und einer ICD-Elektrode, welche zwei Stimulationsringe (Ring 1 und 2) und zwei Schock-Coils (Coil 1 und 2) aufweist. Der Aufbau ist in Abb. 3 dargestellt. Die Elektrode wird substernal implantiert, was eine unmittelbare Nähe zum Herzen ermöglicht. Über die Ringe und Coils sind unterschiedliche Wahrnehmungs- und Stimulationsvektoren möglich, wobei der Standard-Wahrnehmungsvektor Ring 1 zu Ring 2 ist [28]. Zwei weitere Vektoren (Ring 1 zum Gehäuse [Can] und Ring 2 zu Can) ermöglichen alternative Programmierungen der Wahrnehmung bei inadäquaten Messwerten auf dem Standardvektor. Das Spektrum liegt hier bei Werten zwischen 1,86 ± 0,93 [26] und 3,4 ± 2,0 mV [10]. Konsekutiv wird eine höhere Empfindlichkeit als in transvenösen Systemen von bis zu 0,075 mV programmiert.

Zur Stimulation stehen ebenfalls verschiedene Vektoren zur Verfügung, welche drei verschiedene Stimulationsformen anbieten: antitachykarde Stimulation zur Terminierung ventrikulärer Tachykardien ohne Schockabgabe, Post-Schock-Stimulation zur Prävention von Asystolien sowie vorübergehendes antibradykardes Pacing. Die Vektoren zwischen Ring 1 zu Ring 2 und Ring 1 zu Coil 2 sind sog. Low-Output-Stimulationsvektoren, wohingegen Coil 1 zu Coil 2 als High-Output-Vektor mit Amplituden von bis zu 30 V mit einer Impulsdauer von 10 ms stimulieren kann [28]. Die Reizschwellenmesswerte lagen im Mittel bei 10,8 ± 2,2 V bei einer Impulsdauer von 10 ms [5].

### Patientenselektion und Operationsvorbereitung

Die Implantation eines EV-ICD kann in Erwägung gezogen werden, wenn bei Patienten eine primär- oder sekundärprophylaktische Indikation zur ICD-Therapie, jedoch keine dauerhafte Indikation zur Schrittmacher- oder Resynchronisationstherapie besteht [5, 12, 30]. Das Vorhandensein eines elektrodenlosen Schrittmachers stellt keine Kontraindikation dar.

Nicht geeignet sind Patienten mit anderen aktiven Implantaten im Körper (beispielsweise Neurostimulatoren) sowie nach oder vor geplanter Sternotomie. Auch bei Patienten mit ausgeprägten Thoraxanomalien, wie z. B. einem Pectus excavatum oder nach thorakaler Bestrahlungstherapie sollte der EV-ICD derzeit nicht eingesetzt werden.

Der sorgfältigen Operationsvorbereitung und -planung kommt auch beim EV-ICD eine besondere Bedeutung zu [20]. Ein präoperatives EKG-Screening der Patienten zur Eignung für einen EV-ICD ist nicht erforderlich. Eine präoperative CT des Thorax kann zur Planung des operativen Vorgehens mittels Darstellung der anatomischen Verhältnisse hilfreich sein [28]. Hier sind insbesondere die Verhältnisse im Retrosternalraum, die Ausmaße und Lage des rechten Vorhofohres (Risiko des P‑Wellen-Oversensings) sowie das Verhältnis der beiden Lungenflügel zueinander von Interesse [28], da hier die Elektrode implantiert wird [9]. Auch vorhandene Adhäsionen oder Thoraxdeformitäten kommen so zur Darstellung, welche eine Elektrodenplatzierung in diesem Bereich erschweren können [11, 20].

### Implantation

Die Implantation eines EV-ICD kann im Elektrophysiologie-Labor oder Hybrid-Operation erfolgen, wichtig ist das Vorhandensein einer Röntgenanlage mit C‑Bogen. Üblicherweise erfolgt das sterile Abwaschen und Abdecken des gesamten Thoraxbereichs. Ferner erfolgt eine präoperative Antibiotikaprophylaxe. Aktuell ist ein spezielles Training der Operateure sowie bei den ersten 5 Implantationen die Anwesenheit eines Herzchirurgen gefordert.

**Abb. 4:**
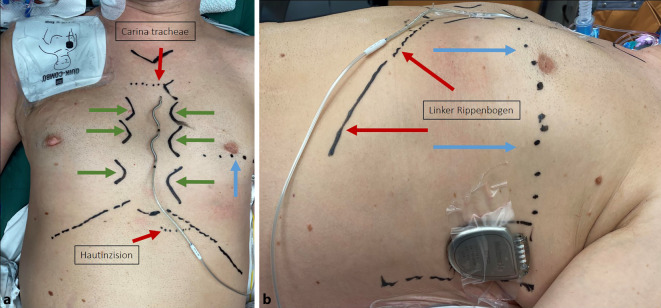
Markierung der wesentlichen Orientierungspunkte von frontal (**a**) und links-lateral (**b**). Es erfolgt eine Markierung des Jugulums und des Proc. xiphoideus, der Sternalgrenzen (*grüne Pfeile*) sowie der Rippenbögen. Ferner ist unter Röntgendurchleuchtung die Höhe der Carina tracheae sowie die Linie zur Markierung der Höhe des linken Herzrandes (*blaue Pfeile*) markiert. Höhe der Sternalgrenzen in *Grün *markiert

Aktuell wird die Durchführung der Operation in Intubationsnarkose empfohlen. Für die ersten Implantationen ist die Anwesenheit eines Herzchirurgen erforderlich. Ein intraoperatives Monitoring der Vitalparameter sowie ein EKG sind obligatorisch, ebenso Defibrillationspatches außerhalb des sterilen Bereichs [11, 28]. Präoperativ erfolgt zunächst eine Markierung der Positionen von Elektrode und Aggregat sowie der geplanten Schnittführung und anatomischen Orientierungspunkte (Abb. 4).

**Abb. 5:**
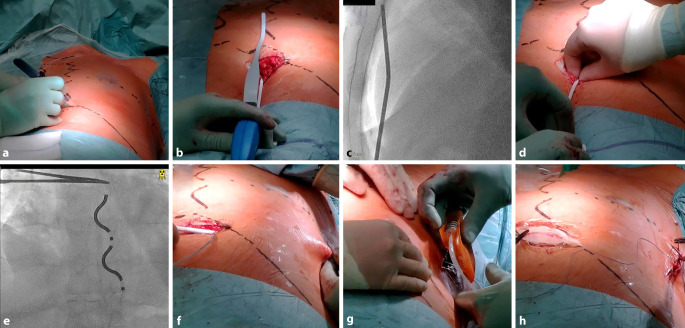
Implantationsschritte bei der Implantation eines EV-ICD. **a** Hautinzision substernal an der zuvor markierten Stelle. Präparation der Rektusscheide und anschließend stumpfe Präparation mit dem Finger in den Retrosternalraum. **b** Einführen des Tunnelierstabs **c** Vorschieben des Tunnelierstabs unter Röntgendurchleuchtung in 90° LAO bis auf Höhe der Carina tracheae. **d** Rückzug der Schleuse zum Freisetzen der Elektrode. **e** Radiologische Kontrolle nach Entfernung der Schleuse. **f** Nach Fixierung der Elektrode Tunnelierung in die laterale Aggregattasche. **g** Konnektion an das Aggregat und Einbringen in die Tasche. **h** Wundverschluss beider Inzisionen

Es erfolgt zunächst eine Inzision unterhalb des Xiphoids von etwa 3 cm zum linken Rippenbogen und die Präparation der Faszie des M. rectus abdominis. Nach Eröffnen der Faszie wird der Retrosternalraum mittels stumpfer Präparation mit dem Finger eröffnet (Abb. 5a, b). Anschließend wird der Tunnelierstab unter Durchleuchtung in den Retrosternalraum eingeführt (Abb. 5b–d). Hierbei ist zu beachten, dass sich die Spitze des Tunnelierstabs immer direkt retrosternal befinden sollte, um das Risiko kardialer oder pulmonaler Verletzungen zu reduzieren. Die Ausrichtung des Tunnelierbestecks wird unter Durchleuchtung in a.-p. kontrolliert, anschließend kann es in lateraler Angulation (90° LAO) langsam vorgeschoben werden. Die Tunnelierung wird unter Kontrolle der Ausrichtung und ohne Widerstand bis auf Höhe der Carina tracheae durchgeführt (Abb. 5e). Nach Entfernung des Tunnelierstabs kann über die Schleuse die Elektrode eingebracht werden. In dieser Position können erste Sensing-Messwerte erhoben werden. Zudem ist auf die Abwesenheit von P‑Wellen zu achten. Beim Auftreten von P‑Wellen kann die Elektrode vorsichtig nach kaudal zurückgezogen werden oder eine Retunnelierung erfolgen. Letzteres ist außerdem bei schlechtem R‑Wellen-Sensing (< 1 mV) notwendig. Ist eine Position mit akzeptablen Messwerten gefunden, erfolgt im Anschluss die Fixierung der Elektrode an der subxiphoidalen Faszie. Die sorgfältige Fixierung mit nichtresorbierbarem Nahtmaterial und einem besonders festen Knoten (z. B. sog. „constrictor knot“) ist von Bedeutung zur Vermeidung postoperativer Elektrodendislokationen.

Wird ein adäquates ventrikuläres Sensing (> 1,0 mV) erreicht, wird anschließend auf Höhe der vorbereiteten Markierung eine subkutane Aggregattasche präpariert. Mithilfe eines weiteren Tunnelierstabs wird die Elektrode in die Aggregattasche hin getunnelt (Abb. 5; f). Nach Konnektion der Elektrode erfolgt anschließend die Fixierung des Aggregats (Abb. 5; g). Eine intraoperative ICD-Testung des EV-ICD-Systems ist empfohlen [9].

Die Operationsdauer von der Hautinzision bis zum Wundverschluss lag in der Zulassungsstudie im Median bei 66 min [15]. Abschließend sollte zwecks Dokumentation der Elektroden- und Aggregatposition ein postoperatives Röntgen-Thorax in zwei Ebenen erfolgen. Ferner ist eine Device-Kontrolle des implantierten Systems am Folgetag unter Evaluation der Sensing-Werte in Rückenlage sowie Links- und Rechtsseitenlage empfehlenswert. Bis zur Device-Kontrolle ist außerdem ein telemetrisches Monitoring sinnvoll. Nach Abschluss der postoperativen Untersuchungen kann eine Entlassung am Folgetag stattfinden.

### Komplikationen

Im Rahmen der Zulassungsstudie trat eine Infektionsrate von 4,1 % innerhalb von 10 Monaten auf [15]. Die meisten Infektionen konnten medikamentös erfolgreich behandelt werden, nur in 1,3 % der Fälle musste das System bei Tascheninfektion explantiert werden. Systemexplantationen erfolgten zumeist aufgrund einer Elektrodendislokation. Inadäquate ICD-Therapieabgaben traten initial bei 9,7 % auf, meist aufgrund von P‑ oder T‑Wellen-Oversensing sowie Noise auf der Elektrode; bevor die Handhabung der Elektroden bei der Implantation während der Studie angepasst und der Algorithmus verbessert wurde. Der Algorithmus „Smart Sense“ wurde in das kommerzielle Gerät des EV-ICD integriert, um inadäquate Schocks durch P‑Wellen-Oversensing zu vermeiden, und erreicht eine Reduktion von 78 %. Im Hinblick auf die adäquaten Therapieauslösungen zeigt sich eine hohe Effektivität mit einer Konversionsrate in den Sinusrhythmus von 100 %. Insgesamt haben sich im Rahmen der Zulassungsstudie sowohl intra- als auch postprozedural nur selten Major-Komplikationen wie beispielsweise Elektrodendislokationen gezeigt [15].

Auch beim EV-ICD können jedoch verschiedene Komplikationen ursächlich dafür sein, dass eine Systemumstellung auf ein transvenöses System erfolgen muss. Im Rahmen der Zulassungsstudie kam es zu insgesamt acht Systemrevisionen, vor allem aufgrund von Infektionen. Vor dem Hintergrund der noch begrenzten Datenlage bezüglich der EV-ICD sollten Patienten im Vorfeld hierüber aufgeklärt werden.

## Fazit für die Praxis

- Bei Patienten mit ICD-Indikation ohne Schrittmacher- und Resynchronisationsbedarf sollte immer an die Option eines nichttransvenösen ICD-Systems gedacht werden.
- Präoperativ sollten die anatomischen Orientierungspunkte sowie die Elektroden- und Aggregatposition (unter Durchleuchtung) markiert werden.
- Ein ausführliches Implantationstraining der Operateure ist sinnvoll und ermöglicht eine hohe Sicherheit im Rahmen der Implantation.
- Die Implantation eines EV-ICD ist in Intubationsnarkose empfohlen, während die S‑ICD-Implantation auch in Lokalanästhesie kombiniert mit tiefer Analgosedierung oder lokaler Nervenblockade möglich ist.
- Der S‑ICD bietet eine effektive ICD-Funktion mit Post-Schock-Pacing über 30 s und guten Langzeitdaten.
- Der EV-ICD bietet zusätzlich die Möglichkeit des ATP und vorübergehender ventrikulärer Stimulation, und die längere Laufzeit des EV-ICD kann die Anzahl von nötigen Aggregatwechseln nach Batterieerschöpfung verringern. Langzeiterfahrungen sind aktuell noch ausstehend.
